# Religious Legal Pluralism in the Shadow of the Centralistic State

**DOI:** 10.1093/ojls/gqaf010

**Published:** 2025-05-07

**Authors:** Chagai Schlesinger

**Keywords:** legal pluralism, law and religion, religious arbitration, Jewish law

## Abstract

Legal pluralism is a useful framework for analysing church–state relationships. Often overlooked, legal diversity also exists *within* religions. This article examines the interactions between the two. It discusses how church–state arrangements influence the internal legal pluralism of religious systems: how religious actors’ depictions of church–state arrangements impact their self-perception of internal legal diversity. Understanding these often-overlooked nuanced and complex influences has descriptive and normative significance. This argument is demonstrated by analysing a case study: the modern transformations of a hyper-pluralistic doctrine in Jewish law, named ‘Kim-Li’. Modern legal centralism rendered rabbinical reluctance towards its application. The article reveals the correlation between rabbinical interpretations of the doctrine and particular assumptions and aspirations regarding church–state structures. By employing legal pluralism/law and religion classifications, the article suggests that reactions to the ‘shadow of the state’ are more diverse and nuanced than the current literature foresees, and concludes by suggesting its rectification.

## 1. Introduction

The analysis of church–state relationships is increasingly theorised through the prism of legal pluralism,^1^ which explores the coexistence of a plurality of normative corpora within the same social environment. From this perspective, the coexistence of communal religious norms alongside official state law raises descriptive and normative questions that the legal pluralism discourse aims to address.^2^ But legal pluralism in the church–state context has yet another dimension. The depiction of two fixed systems—‘religious law’ versus ‘state law’—neglects the pluralistic perceptions of law *within* the respective religious legal systems.^3^ Perhaps due to their pre-modern origins, religions maintaining a dominant normative system^4^ frequently hold a fluid, non-rigid approach towards their own legal practices and traditions.

This article demonstrates how these dimensions of legal pluralism may impact on one another. In particular, it delineates how the ways in which state legal systems construct their relationships *vis-à-vis* coexisting religious law influence the self-perception and practical expression of the legal diversity that these religious systems sustain internally. Different models of church–state relationships, and their different depictions by religious leaders and institutions, affect religious systems’ internal perception and construction of the legal diversity within them. This influence may be subtle, but it is clearly articulated by these religious–legal actors.

In this article, I term these dimensions *external legal pluralism* (indicating legal pluralism between state law and religious law) and *internal legal pluralism* (indicating the formations of legal pluralism within a given religious law).^5^ The present inquiry examines the interactions between external and internal legal pluralism by analysing a particular case study—the internal reforms within a hyper-pluralistic doctrine of Jewish law—and exploring how these were motivated by the anticipated actions of the state.

Beyond analysing the case study itself, the article therefore pursues multiple goals: (i) to demonstrate how church–state arrangements influence the development of religious legal doctrines, in general, and religious internal legal pluralism, in particular;^6^ (ii) to critique, revise and enrich the existing theoretical frameworks on the interactions between state law and non-state-law; (iii) to show how, while these frameworks do go some way towards illuminating the present case study, they simultaneously fail to capture the complexity and variety of religious responses to church–state arrangements; and (iv) to suggest—following the lessons of the case study—a theoretical expansion of the legal pluralistic lens on church–state relationships, thus providing possible paths towards rectifying this inadequacy.

The relationship between the case study and the theoretical frameworks explored here is, thus, reciprocal: the framework facilitates a thicker understanding of the case study and provides tools, conceptualisations and useful research assumptions about it; the case study, in turn, can help refine and perhaps challenge analytical and theoretical claims, thereby contributing to the theory.

The interactions between the internal and external manifestations of legal pluralism stand at the intersection of three scholarly discourses: the legal study of religious law; the study of legal pluralism; and normative inquiries into church–state relationships. Despite these multiple perspectives, the influence of a given church–state model on the internal construction of legal pluralism within religious communities is frequently overlooked. For various reasons (some of which I touch upon here), each discourse tends to focus on one facet of this phenomenon, and conducting a fruitful dialogue among facets is uncommon. Scholarly depictions of religious reactions to state law tend to adopt a statist perspective and centre on power relations, neglecting the nuanced religious doctrines and ideologies that influence this interplay.

To address this gap, the article adopts a multidisciplinary approach, beginning with the doctrinal examination of the religious–legal rule. This analysis is illuminated by overlaying it with recent categorisations from the legal-pluralism and law-and-religion literature to reveal how the doctrinal developments unfolded ‘in the shadow’ of particular church–state relationships as the religious discourse was influenced by the state’s *anticipated* acceptance of its internal legal pluralism.^7^ These insights, in turn, shed light on the categorisations themselves, as they highlight certain surprising means by which church–state models affect the religious law they aim to govern. Thus, the article promotes a trialogue between the research on religious law, on its reciprocal interaction with state law and on the normative consequences of this interaction.^8^

The remainder of this Introduction presents the case study: the modern tale of an old Jewish law doctrine entitled *Kim-Li* (translated literally from Hebrew: ‘I am certain’). Facilitating an extreme form of legal pluralism, this doctrine, which originated in medieval Europe, has met modern rabbinical hesitance. I will explore three 20th-century rabbinical responses to this doctrine, revealing how each one is affected by the shadow of a different vision of church–state relationships.

Jewish law is commonly characterised as presenting robust internal legal pluralism in its jurisprudence and legal practices. The scholarship has elucidated how this religious legal system embraces controversy and plurality of opinion and sustains weak mechanisms for deciding between these opinions. The outcome is a legal system that facilitates the coexistence of contradicting practices.^9^

The Kim-Li doctrine takes Jewish legal pluralism to an extreme. In short, it allows the defendant in a civil trial to argue that, in their opinion, the position of a particular *Halakhic* ruler—a position that fits their case—is the correct one in Jewish law, even if mainstream *Halakhic* scholars rule otherwise. When such an argument arises (typically, in monetary lawsuits), the judge is obliged to defer to the defendant’s position even if he disagrees.^10^

To understand this, consider the following hypothetical illustration: John sues Jill for unlawful trespass. According to the vast majority of *Halakhic* opinions, the act of trespass entitles John to compensation. Imagine, however, that in the entire history of Jewish law, there have been two rabbis who have ruled that trespassing is non-compensable. Jill argues that she follows this opinion and that she is therefore exempt from paying. This is a Kim-Li plea; and, if successful, the judge must defer to it.

While Kim-Li has long fascinated Jewish legal theorists, it has frustrated jurists from a practical perspective. Given the vast plurality of opinion, they have feared that an extensive application of Kim-Li would allow defendants to exploit the legal process and manoeuvre decisions in their favour. Jurists’ objections to it accompanied the Kim-Li doctrine from its very inception, but these objections took on a distinctive shape when Jewish law encountered modernity and legal centralism. Envisioning Jewish law as the basis for a functioning modern state, Zionist rabbis encountered Kim-Li as part of their attempt to modernise and centralise Jewish law.^11^

In this article, I will examine three doctrinal delimitations of Kim-Li during the 20^th^ and 21st centuries, and highlight their motivation to constrain internal legal pluralism for the purpose of adjusting the religious legal system to fit a modern centralist state. My analysis of these delimitations aims to highlight how each one was constructed under a different ‘shadow’ of the state. To this end, I scrutinise these rabbinical responses through two classifications of state-/non-state-law relationships, canvassed respectively by Gila Stopler and Geoffrey Swenson.^12^ I do not assume that these frameworks are the most dominant or influential of their kind; however, as I will explain, they lend themselves to the analysis of the particular case study.

While the extant theoretical frameworks and classifications of legal pluralism contribute to our understanding of the case study, in turn, the case study can help refine and perhaps challenge these classifications and theoretical claims. This fine-tuning could illuminate other cases in which the internal legal pluralism of non-state systems is in transformation. I do not contend that the particular patterns of this case study will duplicate elsewhere. However, I strongly believe we should expect transformations in *any* religious system’s internal legal pluralism to reflect, *inter alia*, that system’s reaction to the state’s construction of their relationship. Hence, after discussing the reciprocal lessons of the case study, I suggest promising contexts, beyond the Jewish–Israeli setting, in which this inquiry should be fruitful.

The article proceeds as follows. Section 2 provides the theoretical background to this study, outlining a three-dimensional analytical framework. I present Swenson’s taxonomy of relationships between state law and non-state law;^13^ Stopler’s taxonomy of intertwinements between church and state;^14^ and the Kim-Li doctrine itself. Section 3 examines three responses to the modern challenge that Kim-Li imposes, and the different assumptions and aspirations that underpin them. Here, we will see how underlying assumptions about the relationality between state law and religious law motivate and frame the religious–doctrinal discussion. Section 4 returns to the theoretical frameworks of legal pluralism and the aforementioned classifications in particular, and explores the lessons the case study can provide for their refinement. Finally, section 5 concludes by reflecting on why this type of analysis is commonly overlooked by the literature and why a multifocal perspective is necessary to overcome it.

## 2. Theoretical Landscape

### A. The Relationality of Legal-Pluralistic Settings

In a recent attempt to outline the core principles of legal pluralism, Ido Shahar and Karin Carmit Yefet highlighted the central role of relationality in this discourse. Relationality, they explain, refers to the idea that ‘the interrelations between coexisting legal orders should not be understood as encounters between well-defined, sealed systems … but rather as encounters between elastic, “porous,” constantly transforming, and easily affected systems’.^15^ Relationality is, indeed, a basic premise of legal pluralism, and scholars from this school are committed to revealing the reciprocal influences of state law and non-state law.^16^ The concept, however, remains under-theorised: beyond *ad hoc* observations of such influences, the literature has generally lacked a comprehensive account of conceptual models of such mutual influence.^17^

To address this gap, Swenson offered four archetypes ‘for understanding the fluid relationship between state and nonstate justice in a wide range of settings’.^18^ First, in the case of *combative* legal pluralism, the state and a non-state legal community are mutually hostile, and each attempts to undermine and, ultimately, destroy the other; under *competitive* legal pluralism, the non-state legal order does not challenge the legitimacy of state law but nevertheless competes with it and strives to encroach on it by increasing its societal influence; *co-operative* legal pluralism refers to a dynamic in which non-state legal actors recognise the state’s normative legitimacy and are willing to collaborate with it to achieve shared goals; and, finally, the *complementary* legal pluralism archetype (the only one of the four that is not relevant to the present study) speaks to the marginal role of non-state law in high-capacity, all-encompassing state legal systems. In these systems, non-state law remains in a strictly complementary role, mainly through private arbitration.

This taxonomy provides useful tools with which to appreciate the tensions within a specific legal-pluralistic setting. Swenson’s goal here is a pragmatic one: any state policy towards non-state legal systems can be evaluated against the landscape of the legal pluralism archetype into which it is placed. However, despite Swenson’s recognition of reciprocity, he focuses on the state’s perspective, discussing several government policies addressing legal pluralism and assessing their ability to promote a shift from one archetype to another.^19^

I adopt Swenson’s classification to assess *relationality* itself: each of the coexisting legal orders has a particular internal perception of the archetypal form that its relationship to the other takes—combat, competition or co-operation. This perception (not necessarily similar for both sides) impacts on the system’s reaction to the other. Moreover, as we will observe, it may also impact on the system’s internal development. Applying Swenson’s taxonomy, then, the question of how the religious system conceives the dynamic of its relationship to state law stands as the backdrop to the present analysis.

### B. Privatisation, Authorisation and Nationalisation of Religion

The relationship between church and state can be depicted as a form of legal pluralism as well. Despite the scholarship’s partial adoption of the term, the evaluation of church–state relationships is usually addressed on a normative scale, by political scientists, political philosophers and legal theorists. Thus, taxonomies of church–state models are somewhat isolated from the legal pluralism discourse. Gila Stopler argues that common descriptive accounts of church–state arrangements are inadequate. She reasons that, while most accounts distinguish between regimes based on their level of separation from/intertwinement with religion—from strict separation to theocracy—they tend to overlook the *particular means* by which these relationships are constructed. This is problematic, argues Stopler, because these means (and not only their outcomes) have important normative consequences. Hence, Stopler suggests we shift our focus from the *extent* of religious entrenchment in state infrastructures to the *methods* by which that entrenchment is constructed—asking ‘how?’ instead of ‘how much?’^20^

On that basis, Stopler proposes a new classification of church–state relationships, based on three categories. The first, *privatisation of religion*, is the classic liberal depiction of separation. Under this category, the state treats religion as a private activity that should not influence public policy, and simultaneously grants it constitutional protection. Second, *authorisation of religion* occurs when the state permits religious entities to execute religious norms as state agents. Third, *nationalisation of religion* refers to scenarios in which the state’s legal infrastructure incorporates religious values or norms into the national legal system. By that, the state has more power to control and regulate the exact scope of religious content. The three categories are not exclusive; they overlap and coexist in many liberal and semi-liberal societies. In analysing the Kim-Li doctrine, to which we turn next, I adopt Stopler’s categories and demonstrate how, historically, each type of church–state relationship distinctively impacted on the internal religious–legal discourse.

### C. The Kim-Li Doctrine: A Short History

Jewish law is typically portrayed as a legal-pluralistic system. Early sources of Jewish law embraced the phenomenon of legal dispute, both jurisprudentially and practically. In addition, Jewish law neglected the development of strong institutional or doctrinal methods for deciding among legal opinions. While Jewish legal pluralism dates back to antiquity, it increased exponentially after the loss of a Jewish sociopolitical centre.^21^

This pluralism, however, did not render Jewish law an individualistic, ‘cherry-picking’ system. Generally speaking, the system came to facilitate the coexistence of several legal *subcultures*, each one applying a slightly different set of norms. The construction, reconstruction and reproduction of these subcultures is a dynamic process,^22^ but the basic notion is that legal pluralism exists among, and is constituted through, legal subgroups or schools.^23^

This infrastructure has important legal implications.^24^ Consider, for instance, religious law’s prohibition on abandoning one’s ancestral familial practices in favour of other practices in the Jewish tradition.^25^ This prohibition contributes to the legal-pluralistic ethos by legitimising and preserving the plurality of practices and traditions within the system. Simultaneously, it reduces *individualistic* pluralism: despite the many opinions and practices that coexist in Jewish law, individuals must adhere to their respective subcultural lineages.

It is within this dynamic that Kim-Li doctrine is applied,^26^ as a defence claim in civil lawsuits. Whenever the mainstream opinions of Jewish law support the plaintiff’s argument but there are also minority opinions that support that of the defendant, the latter can argue, in essence: ‘I hold that the minority opinion is the one we should follow; to force me to pay, you must prove this opinion is wrong; and, since it is impossible for you to do so, I should not be made to pay.’ This, in short, is what is known as a Kim-Li plea.

The argument’s analytical rationale can generally be described as a unique form of a possession rule:^27^ since the defendant possesses the disputed property or payment, it is the plaintiff who carries the burden of proving his or her case. Usually, this refers to proving the facts and the legal basis of their argument. Kim-Li assumes that proving ‘the law’ when it is disputed is impossible—that is, even if the majority of opinions favour the plaintiff, there is no method for ‘proving’ that the minority is not a valid opinion.

The legal-pluralistic foundations of this plea are apparent. It is based on the facts that (i) many undecided legal opinions coexist; and (ii) a judge in a particular case does not hold the authority to enforce one particular opinion (even if it is widely accepted) and order a party to pay according to it.^28^ Modern scholars of Jewish law, celebrating its legal pluralism, have expressed interest in the doctrine.^29^ Note, however, that the pluralism manifested in the Kim-Li plea deviates from that of the subcultural infrastructure described above. Kim-Li enables an individualistic pick-and-choose position that, sporadically and opportunistically, weakens the ability of local courts to constitute communal binding norms. We will see how this tension has played out in different attempts to delimit the doctrine.

Kim-Li’s extreme practical pluralism renders the judicial process unsustainable. Discharging judicial enforcement power merely because other opinions exist is detrimental to a functioning judiciary. Kim-Li received strong opposition on these lines from the very outset,^30^ and, over the years, the rabbinical literature has suggested several structured limitations to be placed on Kim-Li arguments.^31^ However, modernity’s confrontation with Jewish legal pluralism impacted on rabbinical discourse in two related ways. First, the modern notion of legal centralism straightforwardly contradicts the doctrine. Modern visions of law as a comprehensive, uniform corpus regard Kim-Li as a grave deviation from what law is or how it should operate.^32^ Second, this conflict was intensified by aspirations to establish a Jewish state that would base its legal system on Jewish law.^33^ These aspirations were disturbed by Jewish law’s legal pluralism, and by Kim-Li in particular: according to modern perceptions of the law, legal norms must ostensibly be unified, predictable, applied equally and undebatable in court.^34^

Indeed, several 20th-century rabbis and Jewish legal scholars expressed harsh modernistic rhetoric against the Kim-Li doctrine. The most prominent rabbinic example is Rabbi Isaac Herzog (1888–1959). According to Alexander Kaye, Herzog was hostile towards *external* legal pluralism, opposing suggestions of two separate systems—Jewish law and secular law—coexisting in the future Jewish state. Accordingly, he promoted Jewish law as its one-and-only legal system.^35^ It is therefore likely that Herzog would have taken a pro-centralisation stance regarding internal Jewish legal pluralism, too.^36^ His rhetoric on Kim-Li certainly fits this assumption.^37^

Herzog’s opposition to Kim-Li originated in relation to a particular case involving an inheritance dispute following the death of a wealthy Jewish Yemenite, which was litigated in Jewish courts and, subsequently, in British colonial courts. One main facet of the litigation was a Kim-Li argument raised by the possessors of the inheritance. This argument, Herzog described, was scorned by the non-Jewish lawyers, who argued for the parties:

the matter came before their Supreme Court in Bombay … they got tangled up in a Kim Li claim, and it became the talk of foreign lawyers and judges until the expression ‘Kim Li’ became an expression that dishonours our Holy Torah … for many days when foreigners of the above type would meet in the market, they would say to each other ‘Kim Li’ as a joke.^38^

This episode illustrated, for Herzog, the foundational tension between the doctrine and modern jurisprudence. His concerns were not limited to the opinions of non-Jewish lawyers, however: he was also worried at the potentially pernicious impact of this tension on the wider project to have Jewish law adopted as the country’s legal system. In another reflection on the inheritance trial, Herzog concludes: ‘I am sure that if we continue on the same path, we will never succeed in introducing *Hoshen Mishpat* [the civil law code of Jewish law] as the code of the Hebrew state.’^39^ Herzog predicts that, if the Kim-Li doctrine is not extinguished, it will nullify the political efforts to establish Jewish law as the official law of the soon-to-be State of Israel.

The problem was also articulated by non-rabbinical figures advocating for the incorporation of Jewish law into the future state.^40^ In 1938, the journalist Moshe Kleynman wrote:^41^

Regarding civil law, if we are going to want—and surely we are!—to base state law on the foundations of Jewish law, we will face many difficulties if we rule according to the existing doctrines. For example … What modern legal system can accept such a doctrine and what modern state will validate such a doctrine [as] the ‘Kim-Li’ argument?^42^

Legal scholars with similar aspirations repeated this rhetoric once the State of Israel had been established in 1948.^43^ However, despite its appeal, eliminating a long-lasting legal doctrine like Kim-Li was never going to be an easy task. First, the conservative nature of religious law, in general, and Jewish law, in particular, renders legal change a slow, drawn-out process. A complete abolishment of an established doctrine thus seems unrealistic. Moreover, there is a conservative facet embedded within Kim-Li that imposes a further layer upon this difficulty. The doctrine implicitly assumes that even the most learned judges of a given generation are not eligible to decide among the opinions of the great rabbis from earlier times.^44^ Rejecting Kim-Li is therefore tantamount to a judge laying claim to a position that their predecessors did not hold themselves worthy of, which is not a stance easily taken by religious leaders.

Thus, the inherent difficulty of delimiting internal legal pluralism invites us now to examine closely the legal methods that rabbis have used in this process. This examination reveals that the particular legal mechanism and exact scope of doctrinal change were influenced by the rabbis’ depiction of the particular construction of church–state relationships at play. In other words, the borders of internal legal pluralism were designed in the shadow of the state.

## 3. Internal Legal Pluralism in the Shadow of the State: Three Cases, Three Models

This section examines three modern rabbinical responses to Kim-Li. The first two—one presented by Herzog, the other by one of his pupils, Rabbi Binyamin Z Rabinowitz-Te’omim—date back to the 1930s–50s, the formative years of the Israeli church–state relationship. And the third response, from more recent times, derives from private religious arbitration courts that have banned the use of Kim-Li arguments.

The first two responses were fuelled by aspirations of future *co-operation* between state law and religious law: the political–religious ideological viewpoint underlying them was that religious law should be incorporated into state law. Each of the two positions, however, imagined a different type of fusion between internal legal pluralism and modern state centrality. The first operated under a model of the *authorisation* of religious institutions to execute religious law and the second assumed the *nationalisation* of religious law by its incorporation into modern legislation. Aiming towards co-operation, the rabbis who backed such approaches sought to marginalise the internal legal pluralism of religious law. However, depending on the particular model of church–state relationship they had in mind— whether they envisaged the future authorisation or, alternatively, nationalisation of religion—they chose different paths towards the goal of co-operation. On the one hand, authorisation endeavoured to ‘communalise’ state law—that is, to view state-authorised religious law as a new religious legal community or subculture. This artificial community would be legitimised and incorporated into the general Jewish legal-pluralistic infrastructure. On the other hand, nationalisation was an attempt to reconstruct the pluralistic infrastructure by creating a codification of internal legal pluralism, turning it into a systematic, predictable feature of religious law and, thus, satisfying the centralistic tendency of the modern state.

Meanwhile, the third, more recent, response has evolved in a different setting than the previous cases—one in which religious courts are *privatised* and operate *in competition with* the state. The state casts a distinct shadow in this setting, for competition with state tribunals renders particular transformations in religious internal legal pluralism.

### A. Communalising State Law: Authorising a New Religious Legal Subculture

Previously, I emphasised that Kim-Li undermines the communal characteristics of Jewish legal pluralism. It ostensibly enables individuals to impose on the court any *Halakhic* legal opinion that fits their case, regardless of the particular practices of the given community within which the court in question operates. It is not surprising, then, that one strand of delimitations to Kim-Li is based on communal conventions. By and large, this limitation asserts that Kim-Li cannot be raised against a widespread communal practice or custom. In other words, if the given legal subculture or community holds a specific legal opinion to be decisive and practices accordingly, one cannot raise a Kim-Li claim on the basis of a contradicting opinion. Kim-Li, according to this approach, is a valid claim only in legal disputes in which the specific community does not hold a decisive opinion.^45^

This line of delimitation is not readily applicable to the context of incorporating religious law into the state legal system. It addresses the setting of a court in a stable community with clear legal traditions. State law, however, is ostensibly uniform and blind to religious–legal subcultures and particular communal conventions. An attempt to reduce the internal legal pluralism of Jewish law for the sake of its incorporation by the state cannot rely, so it seems, on delimiting its power *vis-à-vis* particular customary conventions. As a matter of reality, the Jewish population in Palestine in Herzog’s time was composed of various subcultures holding to diverse legal practices.^46^ Therefore, that Kim-Li might be delimited by relying on a notion of pre-existing communal customs seems unlikely. Nevertheless, this was the doctrinal method applied by Herzog in his judicial response to Kim-Li.^47^

The rabbinical judicial system under the British Mandate (today, the Israeli Rabbinical Court system) began to formulate in the first decades of the 20th century.^48^ Herzog began to lead this system in 1937, when he was elected the Ashkenazi Chief Rabbi. His treatment of Kim-Li as reflected in actual court decisions was much more conservative than the harsh rhetoric against it for which he is known. In numerous cases, he examined Kim-Li arguments as a matter of routine, without expressing any particular unease.^49^

Nevertheless, Herzog fought vigorously against Kim-Li arguments in one important legal field: in issues regarding the status of women in Jewish law. Herzog’s position (alongside his Sephardic Chief Rabbi counterpart Ben-Zion Uziel) concerning women’s rights and status is well acknowledged in the literature. This effort included attempts to decrease the internal legal pluralism on the matter. In several issues, the two rabbis sought to establish one opinion—the one favouring women’s equality—as *the* binding legal opinion in rabbinical courts.^50^ As Elimelech Westreich observed, this also affected Herzog’s treatment of Kim-Li arguments when they harmed women’s rights.^51^ Kim-Li arguments raised by husbands attempting to exempt themselves from marital obligations were repeatedly rejected in Herzog’s courtroom.

Westreich accurately pinpoints the significance of the method and rhetoric of this doctrinal move. Herzog and Uziel^52^ introduced a new term, ‘custom of the courts’, implicitly invoking the aforementioned approach under which Kim-Li would be unable to overcome an established custom. Regarding at least two issues, they declared one of the contradicting legal opinions as the customary one—stating that it was the custom of the local courts to follow that opinion. This allowed them to reason convincingly that Kim-Li arguments aiming to bypass it should fail. For example, concerning a monetary issue, they wrote that ‘[these] days, it is the custom of all courts to rule against’^53^ the opinion that advantages the husband; and, as a result, they dismissed a Kim-Li argument by stating that ‘according to the custom of the courts in the land of Israel, we cannot exempt the husband … based on a Kim-Li argument’.^54^

Several aspects of this move are worth noticing. The first is the unique characteristic of this new legal community and its asserted customs—a community of courts. This is strikingly different from the realistic, common depiction of the religious community. Indeed, some of the *real* Jewish communities adjudicated by the new rabbinical judiciary did *not* follow the legal opinions that Herzog and Uziel embraced.^55^ But, by arguing that there was a custom *of the courts*, Herzog and Uziel were leveraging the *institutional* centralisation of Jewish law (the establishing of a sole official rabbinical judiciary) to create a *substantial* centralisation—enhancing one legal practice as a custom of these courts and arguing that this custom blocked a pluralistic Kim-Li plea.

Importantly, Herzog and Uziel were not only shifting from actual communities to an imaginary judicial community; they were also limiting it to the *official* rabbinical courts that they were leading and constructing to constitute a modern judicial system.^56^ In other words, they were not only creating a community of courts here, but also one with specific institutional significance.

Moreover, even within the official religious courts, it is unclear how genuine Herzog and Uziel’s description of a widely accepted custom on the matter was. Customary law is a fluid concept, and defining a custom is a complex and easily biased task.^57^ The official rabbinical courts were established shortly before these legal opinions were written, and it is quite far-fetched to argue for the development of a designated custom during this short period. Moreover, this was probably not the only practice in the courts in the 1940s.^58^ This ambiguity enabled the two rabbis to assert the existence of a custom without the associated costs of a radical change in Jewish legal doctrine.

Last but not least, they used the ‘custom of the courts’ as a form of binding precedent. Jewish law does not include any doctrine of a judicial binding precedent,^59^ but it does emphasise the importance of following the tradition of one’s subculture.^60^ By creating an artificial collective—the official rabbinical courts—and alleging a (perhaps fictional) custom of this collective, Herzog and Uziel were able to covertly centralise the legal norms of this novel judicial system.^61^

These events suggest that the method adopted by Herzog and Uziel to diminish Kim-Li arguments in their courts had radical ramifications for the courts’ institutional centralisation. However, while this method impacted on the scope of legal pluralism within the official religious institutions, it did not aim to transform the overall legal-pluralistic *infrastructure* of Jewish law. Let me explain this point.

In his rhetorical attack against Kim-Li, Herzog was concerned that its extreme pluralism would be an obstacle to having Jewish law adopted as state law.^62^ In practice, however, his (quite limited) legal approach to narrowing it did not challenge the pluralistic foundations of Jewish law: it was based on a merely descriptive argument (‘this is how our courts rule’) accompanied by an accepted normative one (‘customary law blocks Kim-Li’). Despite its novelty, Herzog’s move was therefore quite conservative *vis-à-vis* the broad legal-pluralistic paradigm. It was not hostile to the pluralistic nature of Jewish law *per se*; yet, it defeated the potential *individualistic* hyper-pluralistic consequences of Kim-Li by enhancing the widely accepted *communal* form of legal pluralism, adding to this communal infrastructure another legal subculture—that of the state religious institutions.

This insight has important ramifications. First, while Herzog’s method seeks to contribute to the unification of state-based religious law, it does not make any claims for its supremacy over coexisting Jewish legal subcultures. Religious state law is constructed as one community that develops customary law and enforces it through state-based religious institutions, but other communities may thrive elsewhere. At least in this context, Herzog is not occupied with a utopian vision in which Jewish law, as a whole, is centralised. On the contrary, he is taking advantage of Jewish law’s existing pluralism to establish another legitimate (albeit centralised!) legal community. Second, Herzog’s method allows for the creation of a multilayered communal setting in which one can genuinely belong to one religious community and, simultaneously, be legally bound by the practices of another. Perhaps paradoxically, Herzog deepens the theoretical legal-pluralistic infrastructure of Jewish law.^63^

This doctrinal approach to Kim-Li can be conceptualised as a ‘communalisation’ of state law, whereby state-supported religious law is depicted as a distinct Jewish religious community. State-incorporated religious courts are religious actors, operating as a new community, thus creating new communal *religious* legal traditions.^64^ I contend that this conceptualisation is applicable mostly, if not exclusively, to a particular formulation of church–state relations: the *authorisation* of religion.^65^

To understand this position, observe how the subjects constituting Herzog’s novel community are religious *courts*, acting as autonomous religious entities and executing their discretion to apply substantial religious law. This method of incorporating religious law into state law is an act of authorisation, for the state *permits* religious courts to adjudicate religious law and *equips* them with the coercive powers to execute it. Thanks to the authorisation that it grants, the state scarcely needs to interfere in the content of religious law; instead, it delegates this concern to the authorised religious institutions, freeing them to develop it autonomously.

With an authorisation model in mind, Herzog distinguished the official state-supported religious law from non-state religious legal activity by designating it a religious community in its own right. Authorisation enables him to continue treating the religious courts as an organic part of the religious apparatus—as a recognised subculture within it. The result is a state-supported legal system that is able to construct binding uniform customary law without endangering the pluralistic infrastructure of the religious legal system. As such, the former falls short of directly confronting the legitimacy of the Kim-Li doctrine *per se*.

Authorisation, however, is not the only method for incorporating religious law into state law. Stopler juxtaposes it with the nationalisation of religion—the incorporation of religious norms into the state’s secularised legal system by legislation or by entrusting secular courts with religious law’s interpretation and application.^66^ Indeed, the main political strife over incorporating Jewish law into Israeli law in its formative years focused precisely on attempts to nationalise religion. Recent works on Herzog’s legal thought indicate that his main goal was to nationalise Jewish law.^67^ Recall that Herzog’s fear of Kim-Li was related to its detrimental consequences on the attempt to position the Code of Jewish Law as the state’s official code. It is highly doubtful that, in this endeavour, the communalisation method was ever possible. Such an interpretive move would require the recognition of a parliament-enacted legal code, applicable to all citizens and interpreted and executed by non-religious courts, as the religious custom of a new religious community. This interpretation seems unlikely.^68^

Communalising state-*authorised* religious courts to reduce the practical challenges of legal pluralism therefore stood in tension with the (more ambitious) attempt to constitute a modern legal codification of Jewish law. In the next subsection, I turn to an alternative route to delimit Jewish legal pluralism, proposed by an author who, indeed, attempted to codify religious law in a bid to have it adopted by state law, and sought to avoid the consequences of the Kim-Li doctrine for that purpose.

### B. The Religious Nationalisation Project: The Codification of Internal Legal Pluralism

As part of his aspiration to incorporate Jewish law into the country’s legal system, Herzog established a study group aiming to produce modern Jewish legal textbooks, accessible to secular jurists.^69^ The group acted from an understanding that, to adopt Jewish law as the binding law of the country, it had to be suitable for modern lawyers to use. This mission raised several questions that the group members deliberated among themselves and with others.^70^ One of those questions was how to treat the phenomenon of Jewish legal pluralism, or, in other words, how to address instances in which more than one *Halakhic* opinion existed. It is this particular challenge that I foreground in this subsection.

First, it is necessary to explain the innovative feature of this project, compared to previous attempts at codification in Jewish history.^71^ Given its pluralism, collecting and organising the voluminous materials of Jewish law gave rise to myriad Jewish legal texts. However, the gathering and collation of material, in and of itself, did not construct a legal codification in its full modern meaning: an authoritative and binding text addressing all adherents of the legal system.^72^ With one exception,^73^ such an attempt was never made. Take, for instance, the text that is commonly referred to as *the* Jewish legal code: Joseph Karo’s (1488–1575) *Shulhan Arukh*.^74^ This work became culturally analogous to a Jewish legal code, but defining it as a legal codification is anachronistic for several reasons, not least because the book and its commentators regularly express more than one opinion on a given question.^75^ Thus, even though many Orthodox Jews might describe themselves as ‘observing the *Shulhan Arukh*’, these words do not have a defined practical meaning.

Despite the partial features of a legal code this textual precedent embodied, it nevertheless continued to facilitate the pluralistic nature of Jewish law.^76^ Unsurprisingly, however, the rabbinical–political aspirations to incorporate Jewish law into state law cherished such precedents and emphasised the history of codification in Jewish law. The main 20th-century narrators of Jewish legal history constructed it along the lines of a general dynamic tension between pluralism and ‘codifying’ forces. This stance was influenced by the agenda to reformulate Jewish law as a modern legal system.^77^

Against this backdrop, I now return to Herzog and his study group. It is unclear to what extent the purpose of the textbooks this group envisioned was to be adopted as legislative codification or merely to provide jurists with access to Jewish legal material.^78^ Most likely, a gradual vision motivated the project: these books were viewed as a first step in a gradual process by which, eventually, a fully authoritative codification of Jewish law would be written as the state’s legal order. This notion is presented in a booklet printed by the group in 1950, where they set out their working methods.^79^ One of their points addresses the complexity of the internal legal pluralism in Jewish law, and their response to it:

Many details of the law are in dispute and undecided … they require organization and decision: organizing in a short, concise language appropriate for legislation … and deciding between disputed opinions, since we cannot allow a dispute to remain within the law book. We have not yet reached the stage of decision-making. We thus wrote an appendix of doubts we have, to show them to the Chief Rabbi [Herzog] and our other greatest rabbis. For this reason, we sometimes include two opinions in the book, or, if previous rabbis decided the matter, we include the other opinion in footnotes or other sections. We will omit all of that in the advanced stages of our work.^80^

This passage, which constitutes a reflective roadmap, recognises the challenge imposed by internal legal pluralism on the modern codification of Jewish law and postpones its resolution to the distant future. In a letter to the group, possibly responding to the aforementioned ‘appendix of doubts’, Herzog also avoids providing a comprehensive response, writing that the matter requires a future ‘general solution’.^81^

Despite this intentional irresolution, one relatively major output of the study group provided a systematic response to this challenge: a textbook on the laws of sales, written by the group member Rabbi Binyamin Z Rabinowitz-Te’omim and published in 1957.^82^ This text deliberately approached the challenge of internal legal pluralism by offering a unique codification of Kim-Li pleas.

In his introduction, Rabinowitz-Te’omim emphasised that the purpose of the book was to render Jewish law more accessible, and not to serve as an authoritative text for deciding between opinions.^83^ His decisions among opinions in the book were all based, he proclaimed, on the writings of previous rabbis, and, moreover, he invited readers to question his decisions and encouraged them to apply their own discernment. Despite this modest stance, the main body of the text adopted a decisive style, featuring only one opinion on each case.^84^ How, then, did the book present the plurality of opinions that prevailed in Jewish law? Congruent with the programmatic passage we have just seen,^85^ Rabinowitz-Te’omin employed the common rabbinic technique of producing a multilayered text.^86^ Underneath the main writings, which had the appearance of a modern legal code, he added two sections—‘sources’ and ‘annotations’—in which he included alternative legal opinions. This created a visual hierarchy distinguishing between a ‘mainstream’ opinion and marginal ones.

Moreover, Rabinowitz-Te’omim utilised this multilayered style to create a codification of the *implications* of this plurality: the legitimacy of a Kim-Li argument based on marginal opinions. Systematically, regarding each opinion, he codified the pre-existing *secondary* rabbinic literature on the ability to argue Kim-Li based upon it. As he explained in the Introduction:

In the ‘annotation’ section, we included any accepted opinion, even if it is controversial, but it is legitimate to argue Kim-Li based on it; but rejected opinions, usually of only one rabbinic authority … we have only mentioned in the ‘sources’ section—and sometimes we saw the importance of explicitly mentioning that one cannot argue Kim-Li relying on it. We did not arrive at these conclusions by ourselves but, rather, based on precedential rabbinic decisions that we mention.^87^

The textbook was, therefore, a codification not only of the substantial law, but also of the secondary legal discussion around the consequences of the law’s pluralism. Utilising the aforementioned textual multilayering to evaluate the subject of Kim-Li pleas, the author presented only one legal opinion in the body of the text but, in a marginal subsection, ‘accepted’ additional opinions to the extent that they grounded a legitimate Kim-Li argument. Conversely, the opinions he ‘rejected’—those that could not be counted upon even for a Kim-Li plea—were covered in another secondary subsection. A close examination of the text verifies this understanding of the purpose of the book. The phrase ‘Kim-Li’ appears 72 times—almost always referring to marginal opinions and discussing whether a derivative Kim-Li claim should succeed.^88^

Herzog, we recall again, wrote that if the Kim-Li doctrine were to be maintained, ‘we will never succeed in introducing *Hoshen Mishpat* as the codex of the Hebrew state’.^89^ In the case of Rabinowitz-Te’omim, who became one of the main figures of the codification initiative, his fixation on Kim-Li points to anything but a desire to abolish the doctrine. However, his motivations are similar to those of Herzog. Instead of attempting to abolish legal pluralism, Rabinowitz-Te’omin hopes to tame it. By collecting (and discretionally evaluating) all previous rabbinical discussions on the validity of a Kim-Li claim in particular contexts, he creates a codification of internal legal pluralism. It comprises two layers: a primary decision on the substantive main opinion and a secondary decision concerning the ability to challenge this opinion in court. The goal, it seems, is to create a legal code that is predictable, so that, despite its internal pluralism, it may still serve the legal system of a modern state.

This rabbinical response to Kim Li was constructed under the aspiration to constitute church–state relationships via the *nationalisation* of religion. Under nationalisation, the state incorporates religious norms into its legal system by legislation, codification or adjudication. Stopler depicts this method, perhaps counterintuitively, as a means of state control over religion, on the basis that it enables greater state involvement and supervision of religion than the option of authorising religious groups to execute their religious law autonomously. Under this assumption, when intertwining church–state situations arise, it is in the state’s interest to nationalise religion; and, accordingly, religious groups tend to oppose nationalisation and prefer authorisation.^90^

The case of Jewish law in Israel indicates, however, that nationalisation is not a one-directional process. Several Jewish leaders, Herzog predominantly, pushed for nationalisation and rejected a pluralistic system of authorisation, due to their religious and nationalist ideologies.^91^ Such ideologies required religious law to adjust to the modern state infrastructure in various aspects, including redefining the borders of its internal legal pluralism. The *religious* aspiration of nationalisation of religion is carried out, therefore, in the shadow of this project’s ability to succeed. Codifying Kim-Li’s pluralism by creating a legal text that determines whether such an argument is within the legitimate demarcated range of pluralism is an attempt at this reconciliation.

### C. Legal Pluralism in a Competitive Environment: Private Religious Arbitration Courts

We have observed, then, two rabbinic methods for reconciling Jewish internal legal pluralism with the centralistic character of state law. Both methods were motivated by an aspiration, culminating in the 1940s, to co-operatively synthesise the two systems—by establishing state-authorised Jewish courts or, more ambitiously, by incorporating Jewish law into state law.

By and large, this aspirational project failed.^92^ Today, religious law is, indeed, predominant in Israeli law, but not in the all-encompassing form these rabbis envisioned. Religious law was adopted as the official law in just one designated area, albeit an important one: family law. In this area, religious courts were authorised by the state to impose substantive religious law on their religious group members.^93^ Their jurisdiction, however, is limited to issues of marriage and divorce, and they are unauthorised to adjudicate any other kind of dispute.^94^ In almost all other aspects, the Israeli legal system functions as a secular one, in which religious law, in and of itself, holds no official position.^95^ Herzog’s political vision remains, for his contemporary followers, a messianic utopia.

Hence, in contemporary Israeli society, religious communities that want to practice and adjudicate their religious law in areas that extend beyond family law must rely on private institutions operating under arbitration law.^96^ In contrast to the two previous responses hitherto discussed, these courts act *in competition* with state law. According to Stopler’s taxonomy, these courts operate under the *privatisation of religion* paradigm—treating religious legal institutions as belonging to the private sphere.^97^ In this section, I turn next to analyse the impact of this paradigm on the dynamics of internal legal pluralism. *A priori*, privatisation might be conceived as releasing religions from the impact of state law. Counterintuitively, however, we will observe how its competitive nature leads to the strongest effect of state legal centralism on the behaviour of religious actors.

At this point, it is important to distinguish between two types of private religious courts. The first comprises courts pertaining to insular, usually ultra-Orthodox, communities. These courts serve only their community members, who obey them due to religious beliefs and social pressures. In these cases, it is plausible to assume that competition with state law exerts only a marginal effect on their adjudication.^98^ The second type, on which I focus here, is that of religious arbitration courts that serve the general public, aiming to create a religious alternative to secular law. Part of their motivation is, indeed, a religious one—a mission to persuade the public to use religious law instead of the non-religious system. Another aspect of it, perhaps, stems from the old utopia—a belief that, in the future, religious law will prevail and that the foundations for this revolution should be laid.^99^ These courts oppose the pluralistic agenda promoted by Kim-Li and take action to abolish its use.^100^

In a comprehensive work, Adam Hofri-Winogradow analysed the religious mission underlying private religious–Zionist courts, which includes a notion of constructing an alternative to state law.^101^ Hofri-Winogradow documents the hesitancy of the religious leaders of these courts regarding the Kim-Li doctrine. In interviews he conducted during 2008–09, leadership figures expressed their intention to formulate uniform rulings within their courts, to attract litigants and to gain trust as reliable arbitration fora in the eyes of state courts. Since Kim-Li is an obstacle to this goal, they expressed hostility to it in these interviews.^102^

In more recent years, this hostility has taken a new form. First, the issue preoccupied rabbinical writings, with several rabbis from these private systems having written in internal rabbinical journals about the importance of limiting Kim-Li arguments.^103^ Next, private religious courts began to officially ban the parties from raising Kim-Li pleas. In 2021, as part of the aspiration to create a uniform rabbinic alternative to state civil law, religious–Zionist arbitration courts established an umbrella organisation, *Lechatchila*.^104^ Under the slogan ‘Connecting together to the original Israeli law’, their desire to appeal to the public and create an alternative to secular Israeli law is explicit. Notably, the organisation has recently reformed its arbitration agreement, where the first clause now proclaims that ‘the court is not bound to the rules of Kim-Li’.^105^

The proposal for this move was made by rabbis,^106^ and its acceptance was preceded by a public panel on the subject at the organisation’s annual conference in 2022.^107^ Listening to the discussion, two themes emerge: one relates to the effect of Kim-Li on the public and lawyers, and the other concerns its impact on state courts. Regarding the public, the panellists emphasised the fear that the lack of legal certainty caused by Kim-Li would rule out the religious arbitration option in the minds of potential litigants. The other point they raised was the concern that courts, when reviewing religious arbitrators’ decisions according to arbitration law, would not treat them as final decisions. This is because a Kim-Li-based judgment relies on the defendant’s possession. As we saw earlier, since it is a defence argument, it is limited to situations in which the defendant possesses the debated object/payment. The fact that a Kim-Li-based decision relies on the contingent status of possession might render it not final in the eyes of secular courts.

The use of arbitration agreements to achieve policy goals is not a new phenomenon. According to Jewish law, the parties are required to authorise the court not only for external arbitration law requirements, but also as an internal stipulation of Jewish law, to allow the court to deviate from the strict application of Jewish law if necessary. However, while this stipulation is usually used to equip judges with flexibility in applying the law, here it is innovatively employed to achieve centralisation of the law.^108^

Private religious arbitration in civil matters is part of the *privatisation* scheme,^109^ yet privatisation does not necessarily mean full separation.^110^ This treatment of Kim-Li by private courts demonstrates that privatisation does not, indeed, immunise religious law against the influences of state law. Its competitive aspect urges changes in religious law to uphold this competition. In the case of contemporary arbitration courts, it led to the relatively dramatic transformation in the pluralistic legal doctrine of Jewish law: banning the use of Kim-Li arguments through the arbitration agreements signed by the parties.

## 4. Discussion: Multilayered Lessons

The previous section analysed the Kim-Li case study through different theoretical frameworks of legal pluralism and church–state relationships. These frameworks illuminated how external changes in legal pluralism *vis-à-vis state law* influenced the internal changes in legal pluralism within modern *Jewish law*. Methodologically, while these theoretical frameworks offer critical lenses through which to interpret the case study, the analysis of the Kim-Li example is also instructive in questioning and critically developing or refining these theoretical claims. This discussion section therefore aims to assess these mutual benefits of the analysis and outline their potential contribution.

Legal pluralism terminology is frequently used in two contexts: when conceptualising church–state relationships, and in characterising certain phenomena of religious legal systems such as Jewish law and Islamic law.^111^ These two contexts are usually treated as dichotomously distinct realms. In this article, we have observed legal pluralism in *both* contexts to understand the transformations of religious law in a particular case study. The combination of perspectives illuminates the case study and, simultaneously, suggests that a comprehensive theory of legal pluralism should employ this combined perspective in similar settings. Moreover, the case study demonstrates why a partial perspective is insufficient: focusing only on the external aspect of legal pluralism, and neglecting its relational impact on the internal aspect, leads to inadequate and superficial descriptions of church–state models.

The present analysis of the three doctrinal treatments of Kim-Li builds upon two important insights and contributes to their theorisation. The first is that legal pluralism is prevalent not only between state law and other forms of law (indigenous, religious, tribal, and so on), but also *within* these non-state forms of law. The second insight is that this phenomenon of internal legal pluralism is developed and operated in the shadow of the state. It responds to the structures in which religion (in this case) is entangled with state law or competes with it. Religious law adjusts its doctrines, using various doctrinal methods, to accommodate these intertwined relationships. Thus, the particular type of church–state construction that is applied in a given socio-legal setting may impact on how religious law develops and operates within this setting.

We should not, however, expect the dynamics identified in this case study to replicate themselves in other contexts. The case of Kim-Li is unique in several senses. First, the particularities of Israel as a Jewish state and the aspiration of religious forces to influence its policies might intensify the impact of the state’s shadow. Second, the present discussion has centred on the inceptive era of church–state relationships in Israel. Thus, a major part of the story recounted here was the need to *convince* the emerging state to adopt religious law. Beyond the Israeli context, the impact of a state’s shadow on church–state relationships will likely lessen once these relationships stabilise. Finally, while internal legal pluralism characterises many non-state settings, its particular structures vary.

Indeed, my argument is not that religious legal communities will necessarily react to church–state structures following the same patterns manifest in the Kim-Li case. Rather, I claim that, to arrive at meaningful observations on legal pluralism, we must notice the relationality between the various aspects of this phenomenon. Religious systems’ legal pluralism is influenced by the shadow of state law, and that influence is sensitive to the particular design of the shadow that the church–state relationship in question has created. The Kim-Li case study provides a vivid demonstration of these multilayered and interrelated aspects of legal pluralism, illustrating how the different church–state models trigger different internal–doctrinal responses and stances on the part of religious actors. Similar responses might occur in parallel settings, but their unique religious, cultural or legal characteristics will also probably result in significant differences.

I will devote the final part of this article to the various lessons this case study provides to advance our understanding of religious law, legal pluralism and church–state relationships. It is divided into two discussions. First, I reflect on what the case study can tell us regarding the particular classifications I used to analyse it; then, I discuss its implications for the conceptualisation of legal pluralism as a socio-legal theoretical framework, and suggest contexts in which a new conceptualisation may be productive.

### A. Generalising Kim-Li

#### (i) Authorisation

How should we characterise a religion’s adjustment to its authorisation by the state, as exemplified through Herzog’s depiction of religious state law as a new religious community? Anticipating the possibility of authorisation determined Herzog’s initiative to incorporate state-authorised religious courts into the general paradigm of Jewish legal pluralism. Thanks to that approach, the new modern phenomenon ‘survived’ the chaotic nature of Jewish legal pluralism. Hence, the state-authorised system operated in a (relatively) centralistic manner without confronting (or necessitating reform in) the general pluralistic infrastructure of religious law.

Some aspects of this process are unique to the Jewish–Israeli setting. Certainly, ‘communalisation’ fits the unique communal–subcultural character of Jewish internal legal pluralism. And it is intensified by the formative stage of the state’s shadow, when religious courts were authorised by the British Mandate and their authorisation by the State of Israel was still publicly debated.

Nonetheless, this characterisation has illuminating lessons for other settings marked by religious authorisation. First, it demonstrates how authorisation *does* urge religions to adjust themselves to state modernistic depictions of the law, albeit in a relatively subtle way. This is because authorisation, despite the apparent *carte blanche* it lends to religious actors, is still delimited, conditioned and supervised by the state. The extent of this delimitation is contingent on particular political conditions, but these are factored in by religious players and incorporated into their internal doctrines and reasoning.^112^

This is especially true in contexts of a *struggle for authorisation*, when religious entities press the state to authorise them to act under the auspices of state capacity. When authorisation is still in its formative stage and its exact scope is unclear, religions have stronger incentives to adjust their institutional structures to gain such authority. In our case, Herzog’s communalisation was explicitly motivated by the fear that, without its centralising effect, the project of incorporating Jewish law into the state legal system would fail.

It is therefore inaccurate to assume that authorisation will inevitably lead to conservatising and radicalising religious law.^113^ This assumption takes for granted that religious autonomy or the holding of governmental authority releases religions from external pressure. While this might be true in some scenarios, it is not an inherent feature of authorisation. Moreover, authorisation holds a potential *advantage* for promoting religious reform precisely because of the subtle, evolutionary manner in which it operates. The authorisation model enabled Herzog to offer a significant change in the pluralistic nature of Jewish law, focusing on its state-based functioning, without committing to a broader revolution in the structure of Jewish legal pluralism. Indeed, ultimately, Herzog’s ‘custom of the courts’ made a strong impact on the centralisation of rabbinical courts in Israel, while the more radical attempt at codification by his student, Rabinowitz-Te’omim, made no impact at all on Israeli law. To generalise this point, authorising religious actors to act in their genuine, communal capacities but still as state agents might (counterintuitively) allow them to endorse changes in religious law that exceed their internal religious legitimacy under a nationalisation model or their interest under an insular, privatisation model.

#### (ii) Nationalisation

The attempt to nationalise religious law bears important lessons as well. From the state’s perspective, nationalisation may be viewed as a means of controlling religion; however, its *religious* motivations should not be underestimated. Nationalisation might threaten religion because it deprives it of the ability to develop organically and grants this freedom to secular courts or rigid statutory law.^114^ Thus, state attempts towards nationalisation might trigger religious leaders’ political opposition, not their co-operation. In contrast, the formative stage of the Jewish–Israeli church–state relationship reveals the counterpoint. Zionist rabbis were eager to introduce religious law as the binding statutory law, based on their pious beliefs and national identity. They understood that such a project depended on the ability of their law to adapt itself to modern perceptions—both in substance and style. This included embracing legal centralism, which culminated, *inter alia*, in the attempt to codify its legal pluralism. Thus, nationalisation can operate as a secular attempt to regulate religious content, as a religious attempt to incorporate religion into the legal system or, perhaps, as a co-operative process, executed by religious–legislative alliances.

Both authorisation and nationalisation occur, in these cases, under what Swenson describes as *co-operative* legal pluralism—yet, importantly, not necessarily from the state’s viewpoint, but from that of the religious players. The latter express the wish to co-operate with state law to promote their religious meta-ideology, which, accompanied by a realpolitik understanding of its limits, drives them to reform their legal doctrines in the aforementioned ways.

#### (iii) Privatisation

Finally, the story of privatisation also reveals a complicated relationship between law and religion from a religious perspective. The liberal state might prefer the privatisation of religion for its Protestant–individualistic demarcation, but, as Stopler emphasises, the official legal arrangements do not exhaust religion’s influence on public policy.^115^ This is also true *vice versa*: to religious eyes, privatisation might equate to *competition*, and competition can also lead the opponent to adjust some of its policies and agendas. As our case study indicates, the most radical change in Kim-Li jurisprudence occurred in the privatisation context, and exactly for this reason. Under the authorisation or nationalisation models, while, indeed, affected by the shadow of the state, changes might appear more gradually.

None of this means, of course, that religions will always react to state centralisation (or to any other antithetical state legal notion) in predictable ways. The communal infrastructure of Jewish legal pluralism, the nationalist ideologies underlying the Jewish state project or the formative state of these relationships at the time, along with other particularities, all require any comparisons to be extremely tentative and highly context-aware. However, these lessons do reinforce the notion that the analysis of any particular church–state relationship should be sensitive to the impact the relationship has on internal religious discourse and to the fact that such impacts are not one-dimensional.

### B. Taking Relationality Seriously

We have now considered certain lessons that the Kim-Li case provides regarding the conceptualisation of church–state classifications, extending the range of possible religious responses to state regulation. This subsection adopts an overarching perspective on these insights and discusses the contribution of the case study to our conceptualisation and use of legal pluralism as a theoretical framework.

Law-and-religion scholars focus on the *external* meaning of legal pluralism (religion versus state), while scholars of religious law focus on its *internal* meaning, analysing pluralism within the religious community. This article argues that a comprehensive understanding of *relationality*—the idea that coexisting legal worlds reciprocally impact on each other—requires us to account for both aspects. This means that religious law scholars who examine transformations in religious pluralism should consider the shadow of state regulation to be an important explanatory variable of these transformations; and law-and-religion scholars should evaluate the normative significance of this shadow when debating the proper way to design it.

Relationality is frequently overlooked; but, even when addressed, discussions mostly concern the *content* of religious and state norms. Taking relationality seriously leads us to appreciate that coexisting legal systems also influence one another concerning their basic structural and institutional attributes—including, in our context, their treatment of legal pluralism itself. This robust meaning of relationality has potential explanatory power and normative significance regarding other tensions between religious and state law. This article is intended to introduce this theoretical expansion of legal pluralism. Within its scope, it cannot provide a comparative analysis of other cases in which this suggested theoretical framework might be fruitful, for such a comparative endeavour requires a fresh methodology and a separate inquiry. However, suggesting some contexts in which this expanded framework seems productive is worthwhile.

The various interactions between Islamic law and the modern state constitute a prominent candidate for such exploration. The scholarship on Islamic law has observed, separately, its nuanced structures of internal legal pluralism^116^ and its various approaches to external legal pluralism.^117^ Both areas underwent significant transformations during the modern era, and reciprocal interplays between these transformations are perceptible. For example, the efforts of modern Islamic countries to codify their legal systems, at the turn of the 20th century, relied (and impacted) on the ability of religious leaders to reconceptualise the scope of their internal legal pluralism and to enable a selective choice among opinions,^118^ and on a reconceptualisation of their attitude to state law creating such a code.^119^ The theoretical refinement of legal pluralism as framed in this article calls for the examination of the shadow of the particular mechanisms used by the state in adopting religious law via these codifications.^120^

The state’s shadow on Islamic law might also be in play outside a Muslim majority. Islamic minorities, in the UK and elsewhere, have established private arbitration institutions, and their normative legitimacy has been extensively and heatedly discussed.^121^ Does this debate and the struggle for legitimacy impact on the jurisprudence of these institutions? Unlike some scholars, who discuss the possible ramifications for the content of Islamic law that these institutions execute, I suggest inquiring into the impact of competing with state law on the flexibility of legal reasoning. The Jewish case presented an aspiration to decrease legal pluralism for the sake of constituting a modern-looking system, but there are indications of the opposite dynamic in the Islamic case: an *expansion* of this pluralism, to achieve judicial flexibility that can compete with the outcomes of state law.^122^

This inquiry, of course, is relevant to other private religious courts when operating in a minority setting.^123^ In these contexts, it is important to highlight that it might have not only descriptive power, but also normative consequences: the continuous debate on the proper scope of authority granted to religious arbitration panels must take into account the effects of this authority on their adjudication. This is a separate question that requires discussion elsewhere; it highlights, however, the importance of this inquiry for contemporary law making, beyond its theoretical empirical benefits.

My call to take relationality seriously requires, in these cases, close attention to the precise means by which a state regulates religion and to the particular doctrinal change and rhetoric used by religious leadership concerning religious internal legal pluralism. This approach demands that we broaden our viewpoint from the bottom-line outcomes of doctrinal change, and it is my hope that such an inquiry will be developed and inspire new horizons for further research.

## 5. Conclusion: Towards Multifocality

The taxonomy of authorisation, nationalisation and privatisation of religion is a valuable tool with which to differentiate between methods of state regulation of religion. Each method can lead to different responses from religious actors. However, these responses may vary particularly depending on the pre-existing *religious* legal doctrines and traditions, ideologies and political goals at play. Similarly, the taxonomy of combative, co-operative and competitive relations between state and non-state law, while encompassing important aspects of the relationality, should not presuppose that the respective perceptions of these relationships on the part of the state versus the non-state actors are identical, nor what the non-state actors’ goals or ends are in a particular setting.

These dimensions are often overlooked, it seems, because of the detachment between the sociological, the normative and the internal–doctrinal analysis of interactions between religious law and state law. What explains this detachment, and what can rectify it? I would like to sketch some initial thoughts here. Some aspects of this detachment can be attributed to issues of accessibility, academic specialisation and academic culture, but there is more to it than that. One point is the persistent scholarly focus on the *state’s* perspective. Conversations aiming for policy proposals or normative conclusions, such as the ones this article draws upon, naturally focus on state actions and tend to marginalise the religious viewpoint or acknowledge only some of its dimensions. However, choosing between a competitive versus a co-operative archetype of legal pluralism, or between an authorisation and a nationalisation model, is not only for the state to consider; religious actors, too, have their own visions regarding these attitudes. These visions stem from established internal legal doctrines, jurisprudence and ideologies, but they are also cognisant of the political constraints in which they function. Employing a multifocal methodology can enable us to take them into account when seeking to understand the relationality of church–state legal-pluralistic settings.

This raises another important point. Accompanying relationality, a further central element of legal pluralism—and, to a large extent, of law and religion as well—is the concept of *power*.^124^ A sociological examination of coexisting legal cultures invites careful scrutiny of the power dynamics between them—and, indeed, legal pluralism scholarship demonstrates this sensitivity.^125^ The importance of power struggles to the analysis of legal-pluralistic settings should not be underestimated. However, I suggest that we should not reduce this analysis to the power-struggle dimension alone. A ‘power struggle’ depiction of religion and state relationships evokes a legal-realism interpretation of these systems that is interested exclusively in legal outcomes, assuming that each side is merely concerned with increasing its own authority. These premises, however, tend to overlook important doctrinal, cultural and ideological constraints that motivate religious actors. As this article proposes, including an internal–doctrinal analysis of religious law into inquiries on church–state relationships is crucial to our understanding of their impact, both descriptively and normatively.

## Footnotes

1 See eg Russell Sandberg (ed), *Religion and Legal Pluralism* (Ashgate 2015); Rossella Bottoni and others (eds), *Religious Rules, State Law, and Normative Pluralism: A Comparative Overview* (Springer 2016); Michael A Helfand (ed), *Negotiating State and Non-state Law: The Challenge of Global and Local Legal Pluralism* (CUP 2015); Brian Z Tamanaha, *Legal Pluralism Explained: History, Theory, Consequences* (OUP 2021) 116–27.

2 On the descriptive–normative duality of legal pluralism, see eg Norbert Oberauer, ‘Introduction’ in Norbert Oberauer and others (eds), *Legal Pluralism in Muslim Contexts* (Brill 2019) 1.

3 This is not to say that state law is not, in itself, multilayered and pluralistic. See eg Ido Shahar, ‘State, Society and the Relations between Them: Implications for the Study of Legal Pluralism’ (2008) 9 Theoretical Inquiries in Law 417, 441; Tamanaha (n 1) 4–10.

4 Sometimes referred to as ‘religious legal communities’. See Michael Helfand, ‘Religious Arbitration and the New Multiculturalism: Negotiating Conflicting Legal Orders’ (2011) 86 NYU L Rev 1231, 1247.

5 I acknowledge that referring to legal diversity within non-state law as legal pluralism is contestable. Arguably, legal pluralism, as a descriptive construct, requires an advanced degree of legal institutionalism or theorisation that might not fit all cases of religious legal systems. Nevertheless, the term is frequently used in this context by scholars of religious law (Jewish and Islamic). See eg Richard Hidary, *Dispute for the Sake of Heaven: Legal Pluralism in the Talmud* (Brown University 2010). For discussions on Jewish legal pluralist features, see also Hanina Ben-Menahem, ‘Controversy’ in Hanina Ben-Menahem and others (eds), *Windows onto Jewish Legal Culture: Fourteen Exploratory Essays* (Routledge 2011) 3; Menachem Elon, *Jewish Law: History, Sources, Principles* (Jewish Publication Society 1994) 1064–72, 1378–9.

6 The notion that state law influences religious–legal actors is common. See eg Amihai Radzyner, ‘The Impact of Supreme Court Rulings on the Halakhic Status of the Official Rabbinical Courts in Israel’ in Leora Batnitzky and Hanoch Dagan (eds), *Institutionalizing Rights and Religion: Competing Supremacies* 224 (CUP 2017). Usually, however, the scholarship focuses on changes in the substance of religious law to accommodate modern values. My argument, in contrast, focuses on such impacts on the core structure of religious law—that is, its scope of legal pluralism as impacted by the state’s external legal pluralism—and what these influences teach us about the concept of legal pluralism as a theoretical framework.

7 By using the expression ‘in the shadow of the law’, I refer to the well-known notion that official legal arrangements impact on the behaviour of many social actors, even when their actions do not react directly to these arrangements. For the expression’s origins, see Robert H Mnookin and Lewis Kornhauser, ‘Bargaining in the Shadow of the Law: The Case of Divorce’ (1979) 88 Yale LJ 950.

8 The church–state discourse has embraced legal-pluralistic semantics to articulate normative dilemmas, but the substantial contribution to their resolution remains contested. Seemingly, the main potential contribution is providing a nuanced *descriptive* account of religious and state law relationships, on which the *normative* discussion will refine itself. This article promotes this type of contribution. See nn 1–2 above; Ido Shahar and Karin Carmit Yefet, ‘Rethinking the Rethinking of Legal Pluralism: Toward a Manifesto for a Pluri-Legal Perspective’ (2023) LHR 1, 6–7.

9 See eg Hidary (n 5); Ben-Menahem, ‘Controversy’ (n 5); Elon, *Jewish Law* (n 5) 1064–72, 1378–9.

10 Hanina Ben-Menahem, ‘Doubt, Choice and Conviction: A Comparison of the Kim Li Doctrine and Probabilism’ (2003) 14 Jewish Law Annual 3 (2003); Elon, *Jewish Law* (n 5) 1282–3, 1386–7. See also Hanina Ben-Menahem, ‘Postscript: The Judicial Process and the Nature of Jewish Law’ in Neil S Hecht and others (eds), *An Introduction to the History and Sources of Jewish law* (OUP 1996) 421, 431. In Hebrew, see Hanina Ben-Menachem, ‘The Kim-Li Argument’s Historical and Legal Origins’ (1972) 3 Dine Israel 89 [Hebrew] (all translations from Hebrew are mine); Hanina Ben-Menachem, ‘Towards a Jurisprudential Analysis of the Kim-li Argument’ (1980) 6–7 Jewish Law Annual 45 [Hebrew]; Eyal Raz, ‘The Kim-Li Argument in Ashkenazi Medieval Responsa’ (2024) 32 Jewish Law Annual [Hebrew].

11 Indeed, Jewish legal pluralism constitutes a broad challenge for this attempt. For its early articulation, see Izhak Englard, ‘The Problem of Jewish Law in a Jewish State’ (1968) 3 Israel Law Review 254, 267. On the broader challenge of modernity to Jewish pluralism see also the discussion n 34 below.

12 Gila Stopler, ‘Religion–State Relations and Their Effects on Human Rights: Nationalization, Authorization, and Privatization’ (2017) 6 OJLR 474; Geoffrey Swenson, ‘Legal Pluralism in Theory and Practice’ (2018) 20 International Studies Review 438. I do not necessarily argue for the accuracy or comprehensiveness of these models, but merely recognise them as relevant taxonomies for this case study.

13 Swenson (n 12).

14 Stopler (n 12).

15 Shahar and Yefet (n 8) 9.

16 See the examples in ibid.

17 Swenson (n 12) 441 (and see his survey of previous literature). Swenson does not use the term ‘relationality’, but addresses the same phenomenon.

18 ibid 442.

19 For example, Swenson discusses incorporating non-state law into the legal system, subsidising the official system to render it more affordable for litigants, or repressing non-state legal practices by outlawing them. ibid 446.

20 Stopler (n 12).

21 See eg Hidary (n 5); Ben-Menahem, ‘Controversy’ (n 5); Elon, *Jewish Law* (n 5) 1064–72, 1378–9.

22 Their distribution is primarily along geographical/ethnic lines, but can also be based on religiosity or ideology. cf Robert Cover, ‘Foreword: Nomos and Narrative’ (1983) 97 Harvard L Rev 4.

23 The archetype of this infrastructure is probably the example of the houses of Hillel and Shamai, from antiquity. See eg Moshe Halbertal, *People of the Book: Canon, Meaning, and Authority* (Harvard UP 1997) 45–54; Hidary (n 5).

24 For example, it constitutes a legal field of (internal) conflict of laws. See Menachem Elon, ‘Conflict of Laws’ in Menachem Elon (ed), *The Principles of Jewish Law* (Keter Publishing House 1974) 715.

25 See Elon, *Jewish Law* (n 5) 893–4.

26 The precise history of the doctrine is debatable. See the sources n 10 above, and especially Raz (n 10).

27 See Isaac Herzog, ‘Possession in Jewish Law’ (1930–31) 5 Temple LQ 598, 610–11.

28 As mentioned, the lack of judicial power may be understood both in terms of the assumption that all legal opinions are equally legitimate and in terms of a conservative appreciation of later generations’ capacity to decide for themselves. Therefore, one can accept the general pluralistic infrastructure but still embrace judicial discretion to decide which opinion to apply in a given case.

29 Especially Hanina Ben-Menachem, who expressed interest in Jewish legal pluralism in general. See his works cited in nn 9–10 above.

30 On early opposition to Kim-Li, see Elon, *Jewish Law* (n 5) 1282–3 and especially fn 180; Ben-Menachem, ‘Towards a Jurisprudential Analysis’ (n 10) 2 fn 3.

31 For a detailed rabbinical survey, see Ezra Basri, *Dinei Mamonot*, vol 4 (Mechon Haktav 1982) 134–208 [Hebrew].

32 See Tamanaha (n 1) 4–10.

33 This political aspiration, usually referred to as the Hebrew Law Movement, was shared by religious leaders and secular nationalists (notwithstanding obvious tensions between them). See eg Assaf Likhovski, *Law and Identity in Mandate Palestine* (University of North Carolina Press 2006) ch 6; Amihai Radzyner, ‘Jewish Law in Pre-State Palestine’ in Christine Hayes (ed), *The Cambridge Companion to Judaism and Law* (CUP 2107) 317; Alexander Kaye, *The Invention of Jewish Theocracy: The Struggle for Legal Authority in Modern Israel* (OUP 2020) 60–2.

34 This, of course, imposed a broader challenge on Jewish legal pluralism. I focus specifically on the Kim-Li doctrine because it was ‘demonised’ by rabbis as antithetical to the correct functioning of a modern legal system. Attempts to centralise Jewish law were prominent in institutional–procedural contexts; see Kaye (n 33) 99–121; Radzyner, ‘Jewish Law’ (n 33) 332. The treatment of Kim-Li is therefore part of a wider phenomenon.

35 Kaye (n 33) ch 3.

36 Kaye touches on some aspects of Herzog’s effort toward internal centralisation; see ibid 66–71, 99–113. See also Amihai Radzyner, ‘How Much of a Centralist and Innovator Was Rabbi Herzog? Comments on The Invention of Jewish Theocracy’ (2022) 25 Jerusalem Rev Leg Stud 173.

37 Herzog’s treatment of Kim-Li was examined in a few Hebrew works: Ratzon Arusi, ‘Rabbi Herzog’s Treatment to the Kim-Li Argument’ in Shulamit Eliash and others (eds), *Masuah Le’Yizhak*, vol 1 (Yad Harav Herzog 2009) 442 [Hebrew]; Moshe Hershkovitz, ‘Harav Herzog as a Jurist’ (PhD dissertation, Bar-Ilan University 2014) 99–100 [Hebrew]; Elimelech Westreich, ‘The Legal Activities of the Chief Rabbis During the Period of the British Mandate: A Response to the Zionist Challenge’ in Avi Sagi and Dov Schwartz (eds), *A Hundred Years of Religious Zionism, vol II: Historical Aspects* (Bar-Ilan UP 2003) 83, 104 [Hebrew].

38 Isaac Herzog, *Teḥuḳah le-Yiśra’el ʻal-pi ha-Torah*, vol 2 (Mosad Harav Kook, Itamar Warhaftig ed 1989) 137–8 [Hebrew]. On the Historical Episode, see Nathan Brun and Amihai Radzyner, ‘The Fights over the Will of “the Rothschild from Aden”’ (2015) 53 Ha’Uma 162 [Hebrew].

39 Isaac Herzog, *Psakim U’chtavim*, vol 9 (Mosad Harav Kook, 1991) 11 [Hebrew].

40 On the non-religious inspirations to revive Jewish law, see n 33 above.

41 On Kleynman, see ‘Kleynman, Moshe’ in *Leksikon Fun Der Nayer Yidisher Literatur* <https://shaneb48.sg-host.com/lexicon/t/1029> accessed 23 February 2025.

42 Moshe Kleynman, ‘Religion and State’ (1938) 7 Moznaim 539, 545 [Hebrew].

43 See Ben-Menachem, ‘Towards a Jurisprudential Analysis’ (n 10) 2 fn 2.

44 For an exemplary investigation of this Jewish conception, see Menachem Kellner, *Maimonides on the ‘Decline of the Generations’ and the Nature of Rabbinic Authority* (State University of New York Press 1996).

45 See Basri (n 31) 186; Westreich (n 37).

46 See eg Isaac B Gottlieb, ‘The Politics of Pronunciation’ (2008) 32 AJS Review 335; Radzyner, ‘Jewish Law’ (n 33) 323; Kaye (n 33) 99. In this setting, Kim-Li-type arguments were raised before the official courts not solely as an opportunistic defence, but also as a genuine plea to have a case adjudicated according to the parties’ original legal subculture. See eg Ben Zion Eliash, ‘Ethnic Pluralism or Melting Pot? The Dilemma of Rabbinical Adjudication in Israeli Family Law’ (1983) 18 Israel Law Review 348.

47 The first to notice and analyse this move was Westreich (n 37).

48 Kaye (n 33) 104–13.

49 Herzog’s decisions were collected in a nine-volume compilation: Isaac Herzog, *Psakim U’chtavim* (Mosad Harav Kook 1989–91) [Hebrew]. Among these decisions, several mention Kim-Li in a mundane, routine manner. See eg 1:12; 1:29; 6:4; 7:80; 7:85; 7:101; 7:109; 7:112; 8:162; 8:167; 8:239; 9:3; 9:6; 9:28; 9:29; 9:39; 9:47; 9:53; 9:58; 9:60; 9:64; 9:67; 9:93–5; 9:107. Almost none of these provides a discussion of the doctrine itself. For one exception, see 8:159.

50 See eg Westreich (n 37); Eliash (n 46).

51 Westreich (n 37) 110.

52 I am referring to both because the court records I have consulted do not mention a personal author; rather, they proclaim the decision of the court.

53 Zorach Varhaftig (ed), *A Collection of Decisions of the Chief Rabbinate Court of Appeals* (Achva Press 1950) 105, 110 [Hebrew]. cf ibid 54 (using slightly different terminology to express the same idea).

54 ibid 168. For other examples of the same phenomenon, see Westreich (n 37) 108–17.

55 Westreich (n 37) 110–11.

56 They could not have reasonably argued that (also unofficial) rabbinical courts pre-dating the official system had held the same ‘custom’. See Westreich (n 37) 116–17.

57 ibid 117–19.

58 ibid 119; this was critically mentioned by their contemporary—see Paltiel Dykan, *Law of Marriage and Divorce* (Yavneh Press 1956) 123–6 [Hebrew].

59 Elon, *Jewish Law* (n 5) 978–86.

60 ibid 893–4; see also text accompanying n 25 above.

61 Westreich (n 37) 119. Importantly, these decisions are provided in the form of an appeal before lower courts, which increases their centralising effect. On this aspect of the rabbinical appellate court, see eg Kaye (n 33) 104–13.

62 Herzog, *Psakim U’chtavim* (n 39).

63 The way I describe Herzog’s move is far from the commonplace understanding of his centralistic ideology, some of which we will examine in the following subsection. Indeed, it does not exhaust Herzog’s attempts at centralisation. See eg Radzyner, ‘How Much’ (n 36). Several explanations can be given for this deviation from his general approach. Possibly, Herzog anticipated a gradual change in this context, and his ‘custom of the courts’ represents an initial stage in the process of centralising Jewish law.

64 This should be distinguished from rabbinical treatments of the new Jewish state and its legal system as a Jewish community, in order to justify its *deviance* from religious law. See Eliav Shochetman, ‘Israeli Law and Jewish Law—Interaction and Independence: A Commentary’ (1990) 24 Israel Law Review 525.

65 Stopler (n 12) 483.

66 ibid 479.

67 See mainly Kaye (n 33); Hershkovitz (n 37); Arye Edrei, ‘A Religious Zionist Dream: Rabbi Herzog’s Vision of Israeli Law’ (2022) 112 Jewish Quarterly Review 620.

68 I am not arguing that such a move is impossible, but, from an internal religious perspective, it seems unlikely. It was, interestingly, offered by an Israeli Supreme Court Justice in an early decision (CA 63/69 *Joseph v Joseph* 23(i) PD 804 (1969)), in the context of Kim-Li. See Elon, *Jewish Law* (n 5) 1788.

69 The initiator of this project was Rabbi Meir Bar-Ilan. The group started to work on this project a few months before the establishment of the State of Israel and continued for several years. See Kaye (n 33) 113–21; Hershkovitz (n 37) 195–221.

70 Mostly regarding laws that contradicted modern egalitarian values (regarding non-Jews, for example). See Kaye (n 33) 114; Hershkovitz (n 37) 195.

71 On this point, see also Kaye (n 33) 113–21.

72 See Jean Louis Bergel, ‘Principal Features and Methods of Codification’ (1988) 48 La L Rev 1073; Edward Fram, *The Codification of Jewish Law on the Cusp of Modernity* (CUP 2022) 4, fn 14.

73 The comprehensive legal code of Maimonides (1138–1204). On Maimonides’ code and its unsuccessful attempt to exclusive authority, see Elon, *Jewish Law* (n 5) ch 34; Moshe Halbertal, *Maimonides: Life and Thought* (Joel Linsider trans, Princeton UP 2013) 164–96.

74 On Karo’s codification, see Elon, *Jewish Law* (n 5) chs 35–6.

75 See Maoz Kahana, ‘A Universe Made of Words: Bibliography and Utopia in the Halakhic Project of Rabbi Yosef Karo’ (2019) 30 Jewish Law Annual 79 (2019) [Hebrew]. See also Chaim N Saiman, *Halakha: The Rabbinical Idea of Law* (Princeton UP 2018) 167.

76 See also Saiman (n 75); Halbertal, *People of the Book* (n 23) 72–81.

77 I am referring especially to Elon, *Jewish Law* (n 5), and his excessive use of the codification framework. In a slightly different context, see Bernard S Jackson, ‘Judaism as a Religious Legal System’ in Andrew Huxley (ed), *Religion, Law and Tradition: Comparative Studies in Religious Law* (Routledge 2002) 34, 36–7.

78 Kaye (n 33) 113–21 decisively describes the project as aiming towards codification; and, indeed, that was the group’s eventual goal. However, as the following quotes demonstrate, their earliest outputs might have been more modest. See also Elon, *Jewish Law* (n 5) 1451–2 (describing the need for a new codification of Jewish law, but one that would maintain a plurality of opinions).

79 The booklet is entitled ‘Legislation According to the Torah: Its Values, Problems and Methods’. It was edited by Rabbi Binyamin Rabinowitz-Te’omim and printed in 1950 [Hebrew]. On the booklet, see Hershkovitz (n 37) 207.

80 ‘Legislation According to the Torah’ (n 79) 10.

81 Herzog, *Psakim U’chtavim* (n 49) 9:107. This letter to the group contains comments on a work on the laws of sales, a work that the booklet refers to as well.

82 Binyamin Rabinowitz-Te’omin, *Hukat Mishpat: Hilkhot Mekhira* (Harry Fischel Institute 1957) [Hebrew]. See also Hershkovitz (n 37) 207–9. The book probably constituted a more developed version of the early work initiated in the study group.

83 Rabinowitz-Te’omin (n 82) 11 (Introduction).

84 In contrast to what is mentioned in the aforementioned quote from ‘Legislation According to the Torah’ (n 79), namely that ‘sometimes’ they included two opinions in the text itself.

85 ‘Legislation According to the Torah’ (n 79).

86 On this practice of multilayering texts, see Kaye (n 33) 116–20.

87 Rabinowitz-Te’omin (n 82) 11.

88 According to an automated tally in the Online Database ‘Otzar Hachochma’ <https://tablet.otzar.org/#/b/602996/p/21/t/1708968288249/fs/0/start/0/end/0/c/1709134846913> accessed 23 February 2025. On average, the phrase appears once every four pages or less.

89 Herzog, *Psakim U’chtavim* (n 49).

90 Stopler (n 12) 480.

91 See eg Edrei (n 67); Kaye (n 33).

92 Kaye (n 33) ch 5; Likhovski (n 33).

93 Stopler (n 12) 484.

94 In the Jewish context, see Rabbinical Courts Jurisdiction (Marriage and Divorce) Law, 5713-1953 (Isr); HCJ 8638/03 *Amir v Rabbinical Court of Appeals* [2006].

95 However, the legislator has incorporated some religious norms into the legal system, and some religious activity is regulated by official institutions. Stopler (n 12) 481.

96 In this regard, the Israeli setting is similar to other Western jurisdictions. For an account of religious arbitration, see Helfand, ‘Religious Arbitration’ (n 4); Michael A Helfand, ‘Arbitration’s Counter-Narrative: The Religious Arbitration Paradigm’ (2015) 124 Yale LJ 2994.

97 Stopler (n 12) 489.

98 This is facilitated by a religious conviction prohibiting participation in non-religious courts—see eg Helfand, ‘Religious Arbitration’ (n 4) 1247.

99 On this distinction, see Adam S Hofri-Winogradow, ‘A Plurality of Discontent: Legal Pluralism, Religious Adjudication and the State’ (2010) 26 Journal of Law and Religion 57, 63–6. This study details the development of non-insular private religious courts. This differentiation resonates, of course, with Cover’s insular–redemptive distinction. See Cover (n 22).

100 This is not the only impact of their competitive dynamic *vis-à-vis* state law. See Hofri-Winogradow, ‘Plurality of Discontent’ (n 99) 66–74; Amihai Radzyner and Tzvi Dorot, ‘“Legal Alternative and Social Vision”: Procedural Aspects in the Private Rabbinical Courts for Monetary Cases’ (2020) 33 Bar-Ilan Law Review 1 (2020) [Hebrew]; Adam S Hofri-Winogradow, ‘The Increase in Legal Pluralism in Israel: The Emergence of Halakhic Civil Courts in the Religious-Zionist Sector’ (2011) 34 Tel Aviv University Law Review 47 [Hebrew].

101 Hofri-Winogradow, ‘Plurality of Discontent’ (n 99); Hofri-Winogradow, ‘Increase’ (n 100).

102 Hofri-Winogradow, ‘Increase’ (n 100) 79–81. The interviewees did not mention any specific solution to the Kim-Li challenge. One assumed that the private courts would develop a uniform practice that would block Kim-Li (similarly to Herzog’s ‘community’); and another mentioned the idea of using the arbitration agreement to delimit the doctrine—a solution that eventually was implemented, as we will see.

103 See eg Rabbi Ratzon Arusi, ‘Application Rules for Kim-Li’ (2019) 123 Emunat Itecha 77 [Hebrew]; Rabbi Hillel Gefen, ‘Deciding Controversies in Our Times’ (2021) 5 Mishpetei Eretz 175 [Hebrew]; Rabbi Moshe Meir Aviner, ‘The Laws of Kim-Li’ (Pesakim undated) [Hebrew] <www.psakim.org/Psakim/File/12822> accessed 23 February 2025.

104 Translated, roughly, as ‘the preferred way of doing it’ <https://lechatchila.com/Lechatchila/> accessed 23 February 2025 [Hebrew].

105 Arbitration Agreement, in *Lechatchila* <https://tinyurl.com/5n86un6h> accessed 23 February 2025 [Hebrew]. This is not the first official arbitration agreement containing this waiver clause. A former umbrella organisation, Igud Batei Din LeMamonot (now merged into the new organisation), has also recently offered arbitration agreements with a similar clause <www.bateidin.org/?page_id=398> accessed 23 February 2025 [Hebrew].

106 See Rabbi Hillel Gefen (with Rabbis Aaron Feldman and Ido Rechnitz), ‘Deciding Controversies in Our Times’ (Mishpetei Eretz Institute 2019) Position Paper No 14 [Hebrew].

107
*Panel on Lim Li—Merkaz Harav Yeshiva 5782 (2022)* <www.youtube.com/watch?v=QLmDXLsYcsA&t=1s> accessed 23 February 2025 [Hebrew].

108 On the rabbinical requirement to sign an arbitration agreement, see Yo’ezer Ariel, *Arbitration Law: Law, Compromise, and the Arbitration Agreement* (Sha’ar Hamishpat Institute 2005) [Hebrew]. This is the first text, to my knowledge, that explicitly suggests using the agreement to avoid Kim-Li. See ibid 195, 263.

109 Stopler (n 12) argues that arbitration in family law should be counted at least as partial authorisation, for several reasons. Most of them, however, are irrelevant to private law arbitration. Indeed, any kind of arbitration that could be enforced by state law is not fully privatised, but the basic paradigm under which these tribunals operate is one of privatisation.

110 Stopler observes how religion potentially influences politics and government policies *despite* its privatisation. ibid 490–1.

111 For a general view, see eg Ido Shahar, ‘Legal Pluralism and the Study of Shariʿa Courts’ (2008) 15 Islamic Law and Society 112; Ron Shaham, ‘Western Scholars on the Role of the “Ulama” in Adapting the Shari’a to Modernity: A Critical View’ in Meir Hatina (ed), *Guardians of Faith in Modern Times: ʿUlamaʾ in the Middle East* (Brill 2009) 171. For an additional context, see Benjamin Schonthal, ‘The Cunning of Legal Pluralism: Negotiating Monism and Pluralism in Buddhist Law’ (2022) 54 Legal Pluralism & Critical Social Analysis 31.

112 Amihai Radzyner emphasised this point: Radzyner, ‘The Impact of Supreme Court Rulings’ (n 6).

113 Stopler (n 12).

114 ibid 480.

115 ibid 489–91.

116 On Islamic internal legal pluralism, see eg Clark B Lombardi, *State Law as Islamic Law in Modern Egypt* (Brill 2006) 13–17; Ron Shaham, *Rethinking Islamic Legal Modernism: The Teaching of Yusuf al-Qaradawi* (Brill 2018) 16–17.

117 Lombardi (n 116) 47–58.

118 Aharon Layish, ‘The Transformation of the Shari’a from Jurists’ Law to Statutory Law in the Contemporary Muslim World’ (2004) 44 Die Welt Des Islams 85; Shaham, ‘Western Scholars’ (n 111). For an important doctrinal discussion, see Ahmed Gad Makhlouf, ‘Continuity and Change of Traditional Islamic Law in Modern Times: Tarjıh as a Method of Adaptation and Development of Legal Doctrines’ (2023) 12 Oxford Journal of Law and Religion 55.

119 See eg Lombardi (n 116) 78–83.

120 For example, it is fruitful to compare these transformations under semi-secular regimes implying nationalisation (via codifications) with those that were conducted under the Saudi regime, which did not codify the law but rather authorised religious judges to apply it. See Frank E Vogel, *Islamic Law and Legal System: Studies of Saudi Arabia* (Brill 2000); Chibli Mallat, *The Normalization of Saudi Law* (OUP 2022).

121 See eg Yvonne Prief, ‘Muslim Legal Practice in the United Kingdom: The Muslim Arbitration Tribunal’ in Norbert Oberauer and others (eds), *Legal Pluralism in Muslim Contexts* (Brill 2019) 12; Michael J Broyde, *Sharia Tribunals, Rabbinical Courts, and Christian Panels: Religious Arbitration in America and the West* (OUP 2017) 181; Rabea Benhalim, ‘Religious Courts in Secular Jurisdictions: How Jewish and Islamic Courts Adapt to Societal and Legal Norms’ (2019) 84 Brook L Rev 2.

122 Ihsan Yilmaz, ‘Inter-Madhhab Surfing, Neo-Ithihad, and Faith-Based Movement Leaders’ in Peri Bearman and others (eds), *The Islamic School of Law: Evolution, Devolution, and Progress* (Harvard UP 2005) 191, 198.

123 See Broyde (n 121).

124 Shahar and Yefet (n 8) 10.

125 ibid.

